# Peak oxygen uptake in Paralympic sitting sports: A systematic literature review, meta- and pooled-data analysis

**DOI:** 10.1371/journal.pone.0192903

**Published:** 2018-02-23

**Authors:** Julia Kathrin Baumgart, Berit Brurok, Øyvind Sandbakk

**Affiliations:** 1 Centre for Elite Sports Research, Department of Neuromedicine and Movement Science, Faculty of Medicine and Health Science, Norwegian University of Science and Technology, Trondheim, Norway; 2 Department of Physical Medicine and Rehabilitation, St. Olav’s University Hospital, Trondheim, Norway; University of Genoa, School of Public Health, ITALY

## Abstract

**Background:**

Peak oxygen uptake (VO_2peak_) in Paralympic sitting sports athletes represents their maximal ability to deliver energy aerobically in an upper-body mode, with values being influenced by sex, disability-related physiological limitations, sport-specific demands, training status and how they are tested.

**Objectives:**

To identify VO_2peak_ values in Paralympic sitting sports, examine between-sports differences and within-sports variations in VO_2peak_ and determine the influence of sex, age, body-mass, disability and test-mode on VO_2peak_.

**Design:**

Systematic literature review and meta-analysis.

**Data sources:**

PubMed, CINAHL, SPORTDiscus^TM^ and EMBASE were systematically searched in October 2016 using relevant medical subject headings, keywords and a Boolean.

**Eligibility criteria:**

Studies that assessed VO_2peak_ values in sitting sports athletes with a disability in a laboratory setting were included.

**Data synthesis:**

Data was extracted and pooled in the different sports disciplines, weighted by the Dersimonian and Laird random effects approach. Quality of the included studies was assessed with a modified version of the Downs and Black checklist by two independent reviewers. Meta-regression and pooled-data multiple regression analyses were performed to assess the influence of sex, age, body-mass, disability, test mode and study quality on VO_2peak_.

**Results:**

Of 6542 retrieved articles, 57 studies reporting VO_2peak_ values in 14 different sitting sports were included in this review. VO_2peak_ values from 771 athletes were used in the data analysis, of which 30% participated in wheelchair basketball, 27% in wheelchair racing, 15% in wheelchair rugby and the remaining 28% in the 11 other disciplines. Fifty-six percent of the athletes had a spinal cord injury and 87% were men. Sports-discipline-averaged VO_2peak_ values ranged from 2.9 L∙min^-1^ and 45.6 mL∙kg^-1^∙min^-1^ in Nordic sit skiing to 1.4 L∙min^-1^ and 17.3 mL∙kg^-1^∙min^-1^ in shooting and 1.3 L∙min^-1^ and 18.9 mL∙kg^-1^∙min^-1^ in wheelchair rugby. Large within-sports variation was found in sports with few included studies and corresponding low sample sizes. The meta-regression and pooled-data multiple regression analyses showed that being a man, having an amputation, not being tetraplegic, testing in a wheelchair ergometer and treadmill mode, were found to be favorable for high absolute and body-mass normalized VO_2peak_ values. Furthermore, high body mass was favourable for high absolute VO_2peak_ values and low body mass for high body-mass normalized VO_2peak_ values.

**Conclusion:**

The highest VO_2peak_ values were found in Nordic sit skiing, an endurance sport with continuously high physical efforts, and the lowest values in shooting, a sport with low levels of displacement, and in wheelchair rugby where mainly athletes with tetraplegia compete. However, VO_2peak_ values need to be interpreted carefully in sports-disciplines with few included studies and large within-sports variation. Future studies should include detailed information on training status, sex, age, test mode, as well as the type and extent of disability in order to more precisely evaluate the effect of these factors on VO_2peak_.

## 1. Introduction

The Paralympic Games are the world’s second largest sporting event, and athletes with 10 different eligible physical impairments [1] participated in 23 summer disciplines in Rio 2016 and will participate in 6 winter disciplines in Pyoengchang 2018 (https://www.paralympic.org/sports). Of these, 16 of the summer sports and 5 of the winter sports disciplines have at least one sitting class. Depending on the eligibility criteria of each sitting sports discipline, athletes with impaired muscle power, impaired passive range of movement, limb deficiency, leg length difference, hypertonia, ataxia and athetosis are allowed to compete (https://www.paralympic.org/sports). Even though performance in all Paralympic sitting sports disciplines is mainly dependent on the work done by the upper body, the physical demands vary within a spectrum from typical endurance sports requiring high aerobic energy delivery over sustained periods to those performed with relatively low levels of displacement and corresponding low aerobic demands [2].

As an indicator of the humans’ maximal ability to deliver energy aerobically, the measurement of maximal oxygen uptake (VO_2max_) is regarded as the “gold standard” [3]. However, during exercise employing relatively low muscle mass, like in upper-body modes, the cardiorespiratory system is not fully taxed and VO_2max_ is rarely reached even in able-bodied participants [4, 5]. In such cases, peak oxygen uptake (VO_2peak_) denotes the highest oxygen uptake reached during exercise to voluntary exhaustion [3] and is a common indicator of peak aerobic energy delivery capacity during upper-body exercise.

In sitting endurance sports with a continuously high physical effort, VO_2peak_ is suggested to be a paramount determinant of performance [6]. Whereas VO_2max_ values are available for elite athletes in a wide range of Olympic sports disciplines [7–10], only one study by Bhambhani et al. [11] provides a general overview of VO_2peak_ values in trained male wheelchair athletes. However, the latter study does not systematically report VO_2peak_ values for the individual Paralympic sitting sports disciplines. A systematic literature review on VO_2peak_ in Paralympic sports disciplines may, therefore, improve the scientific understanding of sport-specific aerobic demands, which is of importance for scientists as well as coaches and athletes. Furthermore, VO_2peak_ values of sitting sport athletes provide clinicians with a framework of what is possible to achieve in terms of peak aerobic capacity when exercising with a given modality and disability. This might be of relevance for providing feedback to their patients once they start engaging in a particular sitting sport activity.

In addition to the sport-specific demands, disability-related physiological limitations also influence VO_2peak_ in athletes with a disability. One study provided absolute VO_2peak_ in well-trained spinal cord injured (SCI) individuals (1.0–1.2 vs. 2.0–2.3 L∙min^-1^ for tetraplegic (TETRA) vs. paraplegic (PARA), respectively) [12]. In the latter study, large differences in VO_2peak_ were found even within the well-trained individuals with different levels of SCI [12]. Whereas the focus in the few previous studies is on the influence of the different levels of SCI on VO_2peak_ [12, 13], there is lack of knowledge on how VO_2peak_ is influenced in Paralympic sitting sports athletes with other common disabilities, such as amputations, spina bifida and poliomyelitis. Furthermore, in the studies that focus on individuals with SCI, an inverse relationship between level of SCI and VO_2peak_ has been shown [14]. One may therefore expect high within-sports variation in VO_2peak_ in Paralympic sitting sports, since they include athletes with a large heterogeneity in disabilities.

Therefore, the purpose of this systematic literature review and meta-analysis was to (i) identify VO_2peak_ values for Paralympic sitting sports, (ii) examine between-sports differences and within-sports variations in VO_2peak_ and (iii) determine the influence of sex, age, body-mass, disability, test-mode and study-quality on VO_2peak_. We hypothesized that VO_2peak_ values would be highest in Paralympic endurance sports with continuously high physical efforts over sustained periods. The lowest VO_2peak_ values were expected in sports with low levels of displacement and sports where athletes with large disability-related physiological limitations, such as athletes with tetraplegia, participate. Furthermore we expected that within-sports variation would be highest in sitting sports disciplines where athletes with a wide range of disabilities are included.

## 2. Methods

We conducted a systematic literature review and meta-analysis in accordance with the Preferred Reporting Items for Systematic Review and Meta-Analysis (PRISMA) guidelines [15]. Additionally, we registered the study protocol a priori in the International Prospective Register of Systematic Literature Reviews (PROSPERO) under the following registration number: CRD42015025134.

### 2.1 Eligibility criteria

Athletes with a physical disability above the age of 15, who were participating in sitting sports, were eligible for inclusion. An athlete was defined as a person who participates “[…] in an organized team or individual sport requiring systematic training and regular competition against others […]”[16] at least on a national level. This rather broad definition may have resulted in the inclusion of some athletes that cannot be considered “elite”. Athletes with a cognitive impairment were not included, since we would have not been able the separate the influence of the cognitive versus the physical disability on VO_2peak_. Studies were included if absolute or body-mass normalized VO_2peak_ values were directly measured in a standardized laboratory setting. Studies that measured VO_2peak_ in a field setting were excluded due to lack of standardization. Only full-text, cross-sectional and intervention studies published in peer-reviewed journals in English, German or French were considered. Abstracts and conference proceedings were not eligible due to lack of detailed reporting of methods and results.

### 2.2 Data sources and search strategy

PubMed, CINAHL (through EBSCOhost), SPORTDiscus^TM^ (through EBSCOhost) and EMBASE were systematically and independently searched by JKB and BB in October 2016 using relevant medical subject headings, keywords and a Boolean search string. The search string combined synonyms and MeSH terms (the latter only relevant for our search in PubMed) of the two parts of the research question: peak oxygen uptake (outcome measure) and sitting athletes with a disability (population) (see S1 Fig). We decided to construct a broad search string to limit the potential of missing out on studies meeting our inclusion criteria. References of the included studies were searched manually and main research groups in the field were contacted for further identification of studies relevant to the research question.

### 2.3 Study selection

After eliminating duplicates articles, the titles were screened by JKB and BB. We only excluded titles that we were certain not to fit in the area of our review topic (e.g. the title being off topic, the title clearly stating that patients/able-bodied participants were investigated, etc.). Studies that did not directly mention VO_2peak_ in their title but were likely to have included it as a secondary outcome measure, were also included. In a second step, the abstracts of studies deemed relevant by title were read. Articles considered relevant by abstract, were then read in full-text. Details on the studies that were included or excluded based on abstract and full-text, and reasons for the excluded studies can be found in attachment S1 Excel file, sheet “study selection”. All disagreements in the selection process were resolved by discussion between JKB and BB. The two reviewers were not blinded to the names of the authors of the included studies. If multiple studies from the same research group included the same data, only the first published study or the study with the most comprehensive information was included.

### 2.4 Data extraction

Data on the sports discipline competed in, the characteristics of the participants (number of participants, sex, age, body mass, type of disability and training status), test mode and peak oxygen uptake (absolute and body-mass normalized VO_2peak_ values) was extracted from the included studies by JKB with BB cross-checking all the data. Where necessary the unit of the training data was converted from minutes to hours and from miles to kilometers.

In the absence of a valid allometric scaling method that is generalizable to athletes with different disabilities [17], we chose to extract and report absolute and body-mass normalized VO_2peak_ values. When studies did not report absolute VO_2peak_ values (L∙min^-1^), these were calculated by multiplying the individual body-mass normalized VO_2peak_ values (converted from mL to L) by the respective participants’ body mass. When body-mass normalized VO_2peak_ values (mL∙kg^-1^∙min^-1^) values were not reported, these were calculated by dividing the individual absolute VO_2peak_ values (converted from L to mL) by the individual body mass (in kg^-1^). When body-mass was not provided, this was calculated by dividing the individual absolute VO_2peak_ values (converted from L to mL) by the individual body-mass normalized VO_2peak_ values. In case of missing individual data, these calculations were not possible and data are not reported accordingly.

### 2.5 Assessment of methodological quality

The quality of the included studies was assessed by JKB and BB with a modified version of the Downs and Black checklist [18]. Modified versions of this checklist have been employed in several reviews in the field of sports science, which also mainly used cross-sectional studies for data retrieval [19–21]. The original checklist comprises 27 items, which are distributed over five sub-scales: reporting (item 1–10), external validity (item 11–13), bias (item 14–20), confounding (items 21–26) and power (item 27) [18]. For the purpose of the present review the following 12 items were included: 1–3, 5–7, 11, 12, 20–22 and 25. The other items were excluded since our review did not focus on interventions or differences between groups, where statistical considerations needed to be made and significance values or power would have been important. The term ‘patient’ was replaced by participant and ‘treatment’ was interpreted in the context of testing as described by Hebert-Losier et al [21]. The ‘source population’ was defined as all athletes with a disability within the respective sports discipline. All items, except item number 5, were rated as ‘Yes’ (1 point), ‘No’ (0 points) or ‘Unknown’ (0 points). For item 5, sex, age, weight, type of disability and training status were considered to be core confounders [17]. Test mode as well as the time of testing within the season were determined to be secondary confounders. Item 5 was scored with 2 points if all core confounders were mentioned. 1 point was scored if 4 out of the 5 core confounders and 1 secondary confounder were explained. ‘No’ or ‘Unknown’ were scored with 0, as described above. As we regarded the core confounders to be sufficiently assessed in item 5, we chose to in more detail address the determination criteria for VO_2peak_ in item 25. As no uniform criteria for the determination of maximal effort exist in a VO_2peak_ test in an upper-body mode, we defined our own minimum criteria. In accordance with Leicht et al. [22], these criteria should be viewed as a way to exclude studies in which maximal effort was clearly not reached rather than to confirm that VO_2peak_ was reached. In case studies ‘Not applicable’ (N/A) was added as a fourth option for items 7, 11, 12, 21 and 22; and items rated as such were excluded from the analysis. The modified version of the Downs and Black checklist used in this literature review can be found in the S1 Table. Quality cut-off points were decided on retrospectively and studies were ranked to be of low (0–5 points), moderate (6–8 points) or good (9–13 points) methodological quality. The level of evidence for each sports discipline was ranked from unknown to strong by combining the quality scores of each of the studies included in the respective discipline (see Table 1). The case studies were excluded from the analysis on level of evidence.

**Table 1:** Criteria for reporting methodological quality and consistency (adjusted from the criteria provided by van Tulder et al.[23]).

Level	Criteria
Strong	Data provided in multiple studies of good methodological quality
Moderate	Data provided in multiple studies of moderate methodological quality OR in one study of good methodological quality
Limited	Data provided in one study of moderate methodological quality
Very limited	Data provided in one study of low quality

### 2.6 Statistics

All data are presented as means ± standard error (SE) and 95% confidence intervals (CI) unless specified otherwise. A meta-analysis, which is defined as “[…] the use of statistical techniques to integrate and summarize the results of included studies.”[15], was performed by grouping together studies that determined VO_2peak_ in the same sports discipline. Sports discipline means were calculated in Microsoft Excel 2016 (Microsoft Cooperation, Washington, USA) by pooling study means by the random effects approach described more in detail by DerSimonian and Laird [24]. In connection to this, TETRA athletes were previously shown to display significantly lower VO_2peak_ values compared to athletes with other disabilities [25, 26]. Therefore, to lower the variation around the mean and to increase the sensitivity of the statistical tests, only the studies where it was possible to remove the VO_2peak_ data from TETRA athletes were included in the pooling procedure. The only exception was wheelchair rugby where all athletes included had TETRA and all studies in this sports discipline were pooled.

Between-sports differences were analyzed in Microsoft Excel by a one-way ANOVA with Tukey-Kramer Q tests to localize pair-wise differences based on study means and pooled study variances. An α level of 0.05 was employed to indicate statistical significance. To investigate the influence of each of the included studies on the VO_2peak_ values presented for the different sports disciplines, leave-one-out sensitivity analyses were performed in Stata 14.2 (StataCorp LLC, Texas, USA). Furthermore, cumulative meta-analyses were conducted to investigate possible VO_2peak_ changes as a function of time for each of the sport disciplines.

A meta-regression was performed in Stata 14.2 to investigate the relationship between absolute and body-mass normalized VO_2peak_ values, respectively, and the following 11 factors (levels of categorical factors are presented in brackets): age, body mass, percentage of men in each study (%Men), percentage of athletes with tetraplegia (%TETRA), paraplegia (%PARA), an amputation (%AMP), spina bifida (%SB), poliomyelitis (%PM) and athletes with other disabilities (%LA), test mode (arm crank ergometry (ACE), wheelchair ergometry (WERG) and wheelchair treadmill (treadmill) and study quality (moderate, good). Studies that provided information on all factors either as group or individual athlete data were used in the meta-regression. Because of too few studies with complete information, individual athlete data was included where the standard error was replaced by the standard deviation of all participants within each respective study. The levels “poling” and “handbiking” for the factor test mode and the level “low” for the factor study quality were excluded from the meta-regression. This is due to these levels providing only few data points for each factor. Baseline levels for dummy coding the two categorical factors test mode and study quality were “ACE” and “good”, respectively. Only factors that significantly contributed to the model and decreased the Tau^2^ estimate were included in the final meta-regression model. Before performing the meta-regression analyses, the variables were checked for multicollinearity.

A pooled-data multiple regression analysis was performed in IBM SPSS Statistics 24.0 (SPSS Inc., Chicago, USA) to investigate the relationship between absolute and body-mass normalized VO_2peak_ values, respectively, and the following six factors (levels of the categorical factors are presented in brackets): age, body mass, sex (male, female), disability (TETRA, PARA, amputation (AMP), spina bifida), test mode (ACE, WERG, treadmill) and study quality (low, moderate, good). Pooled data of studies that provided individual athlete data on all factors was used in the multiple regression analysis. Excluded from the regression analysis were the levels Les Autres and poliomyelitis for the factor disability, and poling and handcycling for the factor test mode. This is due to these levels comprising less than five percent of the data points of these two factors. Study quality was not entered in the multiple regression analysis as a factor due to too few data points with the level “low” and “good”. Baseline levels for dummy coding the three categorical factors disability, test mode and study quality were “PARA”, “ACE” and “good”, respectively [27]. Only factors that significantly contributed to the model and increased the adjusted R^2^ were included in the final regression model. Before performing the regression analyses, the data set was checked for outliers and multicollinearity, and each variable was tested for normality and homoscedasticity of residuals.

Sports discipline was not included in the meta-regression and multiple regression analyses due to multicollinearity with several of the other included factors. Furthermore, only data in the sports disciplines wheelchair basketball, wheelchair tennis, wheelchair racing and wheelchair rugby was included due to too few data points in other sports disciplines.

All figures and tables including information on VO_2peak_ values are arranged according to absolute VO_2peak_ values from highest to lowest values.

## 3. Results

### 3.1 Study selection and characteristics of included athletes

**Fig 1:**
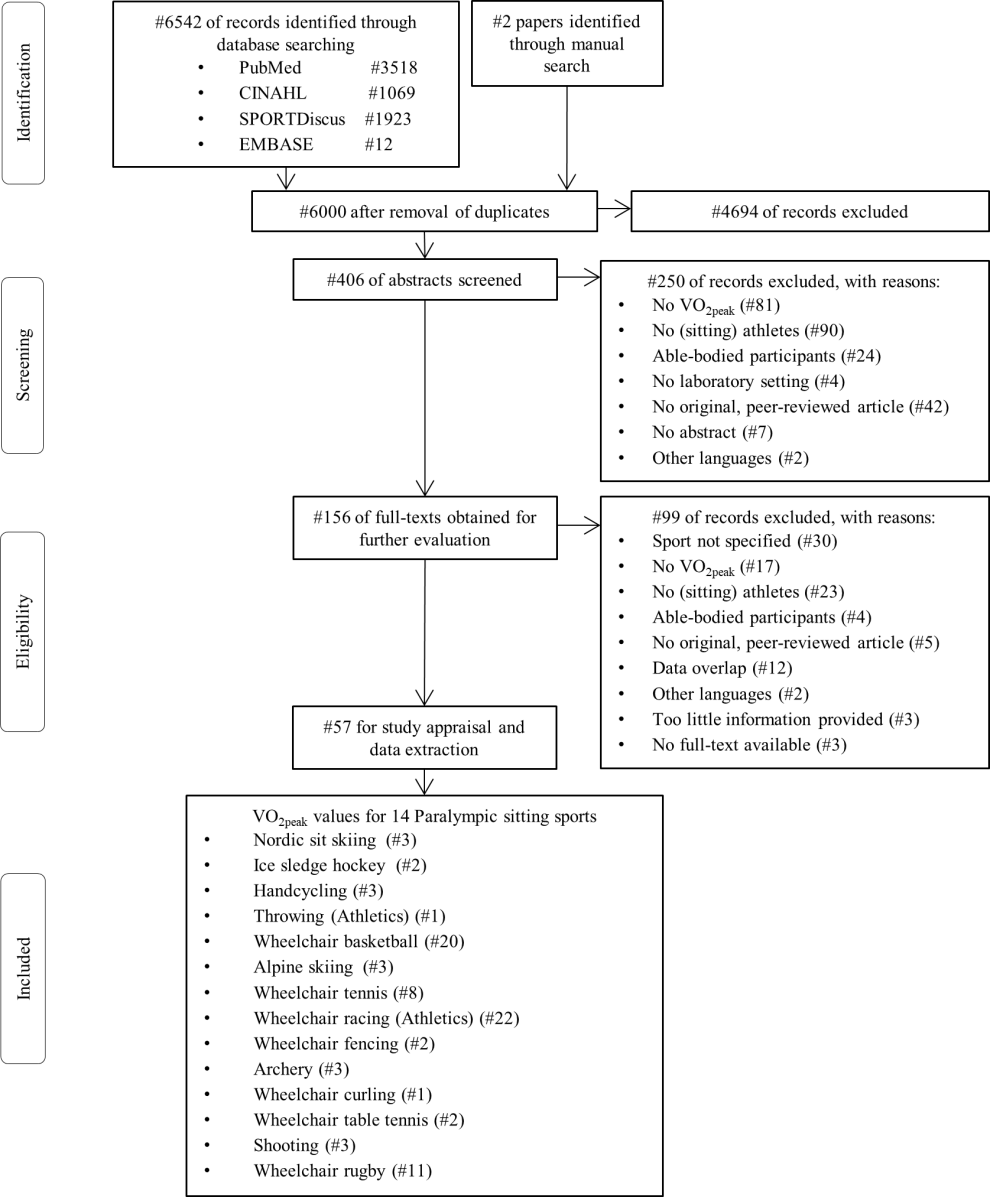
Preferred Reporting Items for Systematic reviews and Meta-Analyses (PRISMA) flowchart depicting the study identification, screening, eligibility and inclusion process. The sports disciplines presented in the box at the bottom are ranked according to their absolute peak oxygen uptake (VO_2peak_) values, from highest to lowest. * Note that 1) some of the studies provide values for more than one sports discipline and 2) athletics was divided into throwing events and wheelchair racing due to the distinct differences in movement demands between these two sub-disciplines.

The systematic search resulted in 6542 studies. After removal of duplicate articles and the subsequent screening process, 57 full text studies were included. These 57 studies reported VO_2peak_ values in 771 athletes from 14 different Paralympic sitting sports disciplines (Fig 1). Athletics was divided into its two sub-disciplines, throwing disciplines and wheelchair racing due to the distinct differences in movement demands. No VO_2peak_ values were reported for wheelchair boccia, para-canoeing, para-equestrian, para-rowing, para-sailing, sitting volleyball, para-triathlon, and para-biathlon.

### 3.2 Methodological quality

Agreement on all assessed quality items was reached by JKB and BB. Four studies were ranked as having low and 6 studies as having good methodological quality (S2 Table). No quality label was attached to the 2 included case-studies. The remaining 45 studies were regarded to have moderate methodological quality. The quality of the studies that are included in each sports discipline determines the level of evidence of the VO_2peak_ values.

### 3.3 Between-sports differences

Mean absolute and body-mass normalized VO_2peak_ ± standard error (SE) of the sports disciplines ranged from 2.9 ± 0.3 L∙min^-1^ and 45.6 ± 5.1 ml∙kg^-1^∙min^-1^ in Nordic sit skiing to 1.4 ± 0.2 L∙min^-1^ and 17.3 ± 3.5 ml∙kg^-1^∙min^-1^ in shooting and 1.3 ± 0.1 and 18.9 ± 1.6 in wheelchair rugby. In Table 2 an overview of absolute and body-mass normalized VO_2peak_ values of all sports disciplines with more than one study with at least 3 participants is provided. In this overview, several factors, such as sex, age, body mass, type of disability, training status and test modes are grouped together. Table 3 and the regression analyses provide details on the influence of these factors on absolute and body-mass normalized VO_2peak_. In the sports with a strong level of evidence and a large number of included studies (wheelchair basketball, wheelchair racing and wheelchair rugby), leave-one-out analyses, examining the effect of each of the included studies, did not have a great impact on neither absolute nor body-mass normalized VO_2peak_ values (S1 Excel file, sheet “MetaInf Output”). However, in sports with a low level of evidence and few included studies, omitting some of the studies had a larger impact on the VO_2peak_ values. With regards to the cumulative meta-analysis, wheelchair basketball and wheelchair racing showed a relatively stable VO_2peak_ over time, whereas wheelchair rugby showed a trend towards an increase in VO_2peak_ (S1 Excel file, sheet “MetaCum Output”). For all other sports, changes over time could not be investigated due to the few number of included studies.

**Table 2:** Overview of absolute and body-mass normalized peak oxygen uptake (VO_2peak_) (mean ± SE [95% CI]) and level of evidence within the separate sitting sports disciplines. Sports disciplines are presented in order of absolute VO_2peak_ values, from high to low.

		Number of athletes	Absolute VO_2peak_ ± SE (L∙min^-1^) [95% CI]	Number of athletes	Body-mass normalized VO_2peak_ ± SE (mL∙kg^-1^∙min^-1^) [95% CI]	Level of evidence
1	Nordic sit skiing	24	2.9 ± 0.3 [2.2–3.5]^WB, AS, WT, WRA, WF, WTT, SH, WRU^	24	45.6 ± 5.1 [35.6–55.6] ^HC, WB, AS, WT, WRA, WF, WTT, SH, WRU^	moderate
2	Para ice hockey	46	2.7 ± 0.3 [2.0–3.3]^AS, WT, WRA, WF, WTT, SH, WRU^	-	-	limited
3	Hand cycling	30	2.6 ± 0.2 [2.2–3.1]^AS, WT, WRA, WF, WTT, SH, WRU^	30	36.0 ± 4.3 [27.4–44.5] ^NS, WRA, WF, WTT, SH, WRU^	moderate
4	Wheelchair basketball	209	2.5 ± 0.1 [2.3–2.7] ^NS, AS, WT, WRA, WF, WTT, SH, WRU^	158	34.5 ± 1.8 [30.9–38.1] ^NS, WRA, WF, WTT, SH, WRU^	strong
5	Alpine sit skiing	21	2.3 ± 0.2 [1.9–2.7] ^NS, PIH, HC, WTT, SH, WRU^	21	33.1 ± 4.8 [23.6–42.5] ^NS, WRA, SH, WRU^	moderate
6	Wheelchair tennis	23	2.2 ± 0.2 [1.8–2.6] ^NS, PIH, HC, WTT, SH, WRU^	23	33.0 ± 2.3 [28.6–37.4] ^NS, WRA, SH, WRU^	strong
7	Wheelchair racing	179	2.2 ± 0.2 [1.9–2.5] ^NS, PIH, HC, WB, WTT, SH, WRU^	110	39.6 ± 1.9 [35.9–43.3] ^NS, HC, WB, AS, WT, WF, WTT, SH, WRU^	strong
8	Wheelchair fencing	10	2.2 ± 0.5 [1.2–3.1] ^NS, PIH, HC, SH, WRU^	10	31.0 ± 3.8 [23.4–38.6] ^NS, WRA, SH, WRU^	moderate
9	Wheelchair table tennis	7	1.8 ± 0.7 [0.5–3.1] ^NS, PIH, HC, WB^	7	29.2 ± 8.7 [12.0–46.3] ^NS, WRA, SH, WRU^	moderate
10	Shooting	8	1.4 ± 0.2 [1.0–1.9] ^NS, PIH, HC, WB, AS, WT, WF, WRA^	8	17.3 ± 3.5 [10.3–24.2] ^NS, HC, WB, AS, WT, WF, WTT^	moderate
11	Wheelchair rugby	114	1.3 ± 0.1 [1.1–1.5] ^NS, PIH, HC, WB, AS, WT, WF, WRA, WTT^	95	18.9 ± 1.6 [15.9–22.0] ^NS, HC, WB, AS, WT, WF, WTT^	strong/moderate

Labels in superscript indicate significant differences to the respective sports discipline

The level of evidence with two attributes refers to absolute/body-mass normalized mean values, respectively. The results of the assessment of methodological quality need to be considered cautiously given the lack of empirical evidence that supports these. Note: several factors such as sex, age, body mass, disabilities, training status and test modes are grouped together in this overview table. Data of athletes with TETRA was excluded from the calculations of all sports discipline means except for wheelchair rugby.

**Table 3:** Data extraction of number of male and female participants, absolute and body-normalized VO_2peak_ values, age, body mass, type of disability, training status, exercise mode, and methodological quality of each of the studies included in this systematic literature review on peak aerobic capacity between and within Paralympic sitting sports. Mean age and body mass ± SE are presented of each sports discipline are presented in the grey lines.

Author and year of publication	Total number of athletes	Male athletes	Female athletes	Absolute VO_2peak_ ± SD (L∙min^-1^)	Body-mass normalized VO_2peak_ ± SD (mL∙kg^-1^∙min^-1^)	Age ± SD (grey lines: ± SE)	Body mass ± SD (grey lines: ± SE)	Disability	Training status	Test mode and protocol	Methodological quality
NORDIC SIT SKIING	24	23	1			41.2 ± 6.3	64.8 ± 5.2				
Bernardi et al. (2010) [2]^‡^	5	5	0	3.3 ± 0.3	51.9 ± 6.9	39.6 ± 7.0	64.6 ± 4.8	3 PARA, 2 PM	*ns*	ACE (R)	good
Bernardi et al. (2012) [6]	16	16	0	2.9 ± 0.5	46 ± 9.8	41 ± 6.7	63.6 ± 6.3	3 AMP, 4 PM, 9 *ns*	*ns*	ACE (R)	low
Bhambhani et al. (2012) [28]^‡^	3	2	1	2.3 ± 0.4	34.7 ± 9.3	44 ± 10.5	67.1 ± 8.8	3 PARA	*ns*	Poling (R)	moderate
PARA ICE HOCKEY	46	46	0			34.1 ± 6.0	75.9 ± 10.5				
Bernardi et al. (2012) [6]	34	34	0	2.5 ± 0.4	32.4 ± 6.1	38 ± 6.8	78 ± 11.4	20 AMP, 2 SB, 2 PM, 1 LA, 9 *ns*	*ns*	ACE (R)	low
Sandbakk et al. (2014) [29]	12	12	0	2.8 ± 0.3	-	28 ± 9.0	74.0 ± 10.0	12 *ns*	491 ± 112 hrs/year	Poling (R)	moderate
HANDCYCLING	30	20	2			41.3 ± 3.8	70.2 ± 3.7				
Fischer et al. (2014) [30]^‡^	12	6	1	2.2 ± 0.6	31.7 ± 8.2	42.4 ± 5.1	68.1 ± 7.5	7 PARA	6.3 ± 2.9 hrs/week	Handbike (R)	moderate
		4	1	2.1 ± 0.6	32 ± 7.1	42.8 ± 4.5	64.4 ± 5.8	5 PARA	6.6 ± 2.6 hrs/week		
Knechtle et al. (2004b) [31]	8	*ns*	*ns*	2.6 ± 0.4	37.5 ± 7.3	38.6 ± 5.9	71.4 ± 8.4	6 PARA, 2 AMP	*ns*	Handbike (S)	moderate
Lovell et al. (2012) [32]^‡^	10	10	0	3.2 ± 0.4	40.4 ± 5.5	40.8 ± 7.6	80.3 ± 7.8	9 PARA, 1 SB	230 ± 57 km/week	ACE (R)	moderate
THROWING (Athletics)	4	4	0								
Gass & Camp (1979) [33]	4	4	0	2.6 ± 0.5	30.1 ± 4.2	-	85.5 ± 9.98	4 PARA	8 ± 4 hrs/week	Treadmill (S-I)	good
WHEELCHAIR BASKETBALL	234	198	36			29.0 ± 1.7	69.9 ± 3.0				
Bernardi et al. (2010) [2]^‡^	13	13	0	2.7 ± 0.5	36.9 ± 3.7	30.8 ± 7.2	73.5 ± 9.3	7 PARA, 4 AMP, 2 PM	*ns*	ACE (R)	good
Bloxham et al. (2001) [34]^‡^^,^^†^	6	6	0	2.6 ± 0.6	37.6 ± 6.7	26 ± 5.9	69.1 ± 9.5	3 AMP, 3 SB	*ns*	WERG (S)	low
Coutts et al. (1990) [35]^‡^^,^^†^	3	3	0	2.6 ± 0.4	34.6 ± 3.9	32 ± 9.5	74.5 ± 15.0	2 PARA, 1 PM	*ns*	WERG (R)	low
Croft et al. (2010) [36]^‡^^,^^†^	6	4	2	3.0 ± 0.9	39.8 ± 5.4	26.7 ± 5.5	74.1 ± 18.2	3 PARA, 1 SB, 2 LA	15.8 ± 3.7 hrs/week	Treadmill (R)	moderate
de Lira et al. (2010) [37]^‡^	17	17	0	1.9 ± 0.4	30.8 ± 6.1	25.4 ± 4.4	63.9 ± 15.4	7 PARA, 2 AMP, 8 PM	*ns*	Treadmill (S)	moderate
Dwyer & Davis (1997) [38]	13	0	13	1.7 ± 0.4	26.8 ± 5.3	26 ± 6.0	62.5 ± 9.5	13 *ns*	*ns*	ACE (R)	low
Goosey-Tolfrey & Tolfrey. (2004) [39]^‡^^,^^†^	1	0	1	1.6	-	22.0	60.0	1 PARA	*ns*	WERG (S)	moderate
Goosey-Tolfrey et al. (2005) [40]^‡^	12	12	0	2.8 ± 0.5	-	32.3 ± 4.6	74.7 ± 14.4	7 PARA, 1 AMP, 2 SB, 2 PM	20 hrs/week	WERG (S)	good
Goosey-Tolfrey & Tolfrey (2008) [41]	24	2	0	2.2 ± 0.2	-	35 ± 1.0	75.8 ± 14.9	2 *ns*	*ns*	WERG (S)	moderate
	-	11	0	2.5 ± 0.2	-	28 ± 5.0	71 ± 8.7	11 *ns*	*ns*	WERG (S)
	-	4	0	2.3 ± 0.1	-	32 ± 3.0	70.7 ± 5.8	4 *ns*	*ns*	WERG (S)	
	-	7	0	3.3 ± 0.3	-	28 ± 7.0	79.2 ± 10.0	7 *ns*	*ns*	WERG (S)	
Goosey-Tolfrey et al. (2014) [42]	17	9	0	2.7 ± 0.5	-	29 ± 9.0	70.3 ± 12.6	9 *ns*	14.9 ± 1 hrs/week	Treadmill (I)	good
	-	8	0	3.8 ± 0.3	-	27 ± 8.0	84.8 ± 10.7	6 AMP, 2 LA	14 ± 3 hrs/week	Treadmill (I)	
Griggs et al. (2015) [43]^‡^	8	7	1	1.9 ± 0.5	-	27.8 ± 6.2	67.7 ± 13.1	8 PARA	16 ± 2 hrs/week	Treadmill (S)	low
Knechtle & Knopfli. (2001) [44]^P^^,^^‡^^,^^†^	10	10	0	2.5 ± 0.4	35.4 ± 4.5	29.4 ± 6.3	72.8 ± 16.9	7 PARA, 1 AMP, 1 PM, 1 LA	*ns*	Treadmill (I)	moderate
	1	1	0	2.8	38.3	21	84	1 TETRA	*ns*	Treadmill (I)	
Leicht et al. (2012) [45]^‡^	9	9	0	2.5 ± 0.3	34.9 ± 5.1	30.6 ± 9.0	71.9 ± 12.6	9 PARA	11.6 ± 4.1 hrs/week	Treadmill (I)	good
Leicht et al. (2014) [46]	9	8	1	2.1 ± 0.5	32.8 ± 10.3	26.2 ± 5.6	64.1 ± 10.4	8 PARA, 1 LA	10.6 ± 5.5 hrs/week	Treadmill (S)	moderate
Rotstein et al. (1994) [47]^‡^^,^^†^	8	8	0	2.0 ± 0.7	26.3 ± 7.5	31.3 ± 9.5	76.1 ± 20.4	4 PARA, 2 AMP, 1 PM, 1 LA	*ns*	ACE (R) /Treadmill (I)	moderate
Schmid et al. (1998) [48]	13	0	13	-	33.7 ± 5.2	27.8 ± 5.6	56.5 ± 6.8	9 PARA, 4 *ns*	7.6 ± 2.1 hrs/week	WERG (R)	moderate
vd Woude et al. (2002) [26]	5	0	5	1.5 ± 0.7	-	30.8 ± 6.3	67.6 ± 18.4	5 LA	8.4 ± 5.5 hrs/week	WERG (R)	moderate
Vanlandewijk et al. (1994) [49]^‡^	40	13	0	1.9 ± 0.5	29.7 ± 8.6	29.6 ± 4.8	65.5 ± 12.6	12 PARA, 1 PM	4.5 ± 1.7 hrs/week	Treadmill (S)	moderate
	-	14	0	2.4 ± 0.4	36.3 ± 9.3	32.9 ± 8.4	70.7 ± 12.4	8 PARA, 1 SB, 5 PM	6.4 ± 3.4 hrs/week	Treadmill (S)	
	-	13	0	2.6 ± 0.3	37.9 ± 5.2	32.8 ± 7.2	67.9 ± 12.2	2 PARA, 3 AMP, 1 SB, 7 PM	5.5 ± 1.7 hrs/week	Treadmill (S)	
Veeger et al. (1991) [50]	11	11	0	2.7 ± 0.6	37.9 ± 6.9	29 ± 3.5	72 ± 9.4	11 *ns*	*ns*	Treadmill (S-I)	moderate
Zacharakis et al. (2012) [51]^E^^,^^‡^	8	8	0	1.7 ± 0.1	-	31.4 ± 8.4	72.8 ± 8.5	1 TETRA, 7 PARA	*ns*	WERG (R)	moderate
ALPINE SIT SKIING	23	21	2			32.2 ± 5.0	61.6 ± 7.3				
Bernardi et al. (2012) [2]	15	15	0	2.3 ± 0.4	31.3 ± 6.7	33.1 ± 4.2	75.9 ± 15.4	1 SB, 14 *ns*	*ns*	ACE (R)	low
Gass & Camp (1979) [33]	3	3	0	1.6 ± 0.5	30.6 ± 9.7	-	52.4 ± 5.3	2 PARA, 1 PM	3.5 ± 2.2 hrs/week	Treadmill (S-I)	good
Goll et al. (2015) [52]	5	0	2	1.8 ± 0.2	44.5 ± 4.9	18.5 ± 0.7	40 ± 0.0	2 *ns*	*ns*	ACE (R)	moderate
	-	3	0	2.4 ± 0.2	35 ± 3.6	31 ± 5.9	69.0 ± 10.0	3 *ns*	*ns*	ACE (R)	
WHEELCHAIR TENNIS	36	29	7			30.0 ± 3.7	64.7 ± 4.9				
Bernardi et al. (2010) [2]^‡^	4	4	0	2.3 ± 0.3	33.1 ± 2.9	38.5 ± 10.3	68.5 ± 8.4	4 PARA	*ns*	ACE (R)	good
Croft et al. (2010) [36]^‡^^,^^†^	6	4	2	2.1 ± 0.7	31 ± 6.6	23 ± 8.2	65.8 ± 18.1	3 PARA, 3 LA	14.7 ± 7.8 hrs/week	Treadmill (R)	moderate
Diaper & Goosey-Tolfrey (2009) [53]	1	0	1	2.0	39.5	33.0	50.1	1 PARA	*ns*	WERG (S)	-
Goosey-Tolfrey & Tolfrey (2004) [39]^‡^^,^^†^	3	0	3	1.7 ± 0.5	32.4 ± 7.0	28.7 ± 5.9	51.0 ± 8.4	3 PARA	*ns*	WERG (S)	moderate	
Goosey-Tolfrey et al. (2006) [54]^E^^,^^‡^^,^^†^	4	4	0	1.0 ± 0.3	14.9 ± 2.6	30 ± 4.3	68.3 ± 7.9	4 TETRA	*ns*	ACE (R)	moderate
Goosey-Tolfrey et al. (2008) [55]^E^^,^^‡^	8	7	1	1.9 ± 0.7	-	27.2 ± 6.9	68.3 ± 17.9	2 TETRA, 3 PARA, 1 SB, 2 LA	*ns*	WERG (S)	moderate
Roy et al. (2006) [56]^‡^^,^^†^	6	6	0	2.1 ± 0.5	27.5 ± 6.5	40.2 ± 9.8	77.5 ± 15.5	5 PARA, 1 AMP	8.7 ± 3.3 hrs/week	ACE (R)	moderate
Vinet et al. (1996) [57]^‡^^,^^†^	4	4	0	2.4 ± 0.2	34.9 ± 1.8	28 ± 5.0	67.8 ± 5.7	4 PARA	4.8 ± 1.0 hrs/week	Treadmill (S)	moderate
WHEELCHAIR FENCING	11	10	1			31.9 ± 4.4	69.0 ± 8.6				
Bernardi et al. (2010) [2]^‡^	6	6	0	2.4 ± 0.7	34.4 ± 5.8	31.8 ± 5.4	68.3 ± 7.0	4 PARA, 1 AMP, 1 PM	*ns*	ACE (R)	good
Veeger et al. (1991) [50]	5	4	0	2.0 ± 0.4	29.2 ± 3.6	32 ± 3.3	70.0 ± 10.2	4 *ns*	*ns*	Treadmill (S-I)	moderate
	-	0	1	1.2	-	-	-	1 *ns*	*ns*	Treadmill (S-I)	
WHEELCHAIR RACING (athletics)	205	177	24			29.0 ± 1.4	61.4 ± 1.8				
Bernardi et al. (2010) [2]^‡^	6	6	0	3.1 ± 0.3	48.1 ± 6.4	30.2 ± 7.0	64.0 ± 7.2	5 PARA, 1 AMP	*ns*	ACE (R)	good
Bhambhani et al. (1995) [58]^E^^,^^‡^^,^^†^	8	8	0	1.4 ± 0.4	19.8 ± 4.3	31.8 ± 6.9	72.1 ± 6.9	8 TETRA	*ns*	WERG (S)	moderate
Campbell et al. (2004) [25]^P^^,^^‡^	20	3	0	1.3 ± 0.2	-	34 ± 8.0	67.5 ± 3.2	3 TETRA	5.4 hrs/week	Treadmill (I)	moderate
	-	8	0	2.1 ± 0.6	-	32 ± 6.0	67.8 ± 7.6	8 PARA	5.4 hrs/week	Treadmill (I)	
	-	9	0	2.2 ± 0.5	-	30 ± 8.0	62.8 ± 10.9	9 PARA	6.0 hrs/week	Treadmill (I)	
Cooper et al. (1992) [59]^‡^^,^^†^	11	11	0	2.6 ± 0.3	39.8 ± 4.2	30.9 ± 6.1	66.0 ± 6.4	10 PARA, 1 SB	7.9 ± hrs/week	WERG (S/R)	moderate
Cooper et al. (1999) [60]^P^^,^^‡^^,^^†^	7	6	1	2.8 ± 0.8	41.0 ± 11.9	31.7 ± 4.9	68.8 ± 6.2	7 PARA	*ns*	ACE (R) /WERG (R)	moderate
	3	1	2	1.4 ± 0.6	22.4 ± 7.6	28.3 ± 2.5	61.2 ± 12.4	3 TETRA	*ns*	WERG (R)	
Coutts & Stogryn. (1987) [61]^‡^^,^^†^	4	4	0	2.7 ± 0.9	41 ± 9.9	26.8 ± 4.4	71.2 ± 16.7	3 PARA, 1 PM	*ns*	WERG (R)	moderate
	2	2	0	1.0 ± 0.02	17.1 ± 0.3	25.0 ± 1.4	59.4 ± 0.3	2 TETRA	*ns*		
Coutts et al. (1990) [35]^‡^^,^^†^	6	6	0	3.1 ± 0.5	52.7 ± 7.8	25.7 ± 4.0	58.5 ± 8.0	2 PARA, 3 AMP, 1 PM	*ns*	WERG (R)	low
Crews et al. (1982) [62]^‡^^,^^†^	4	4	0	2.2 ± 0.1	30.9 ± 7.1	28.8 ± 3.7	73.3 ± 3.7	3 PARA, 1 AMP	72.4 ± 33.8 km/week	Treadmill (S)	moderate
Gass et al. (1979) [33]^P^	4	4	0	2.3 ± 0.6	38.4 ± 9.5	-	61.3 ± 6.5	4 PARA	4.1 ± 1.8 hrs/week	Treadmill (S-I)	good
	1	1	0	1.1	19.4	-	54.6	1 TETRA	1.5 hrs/week		
Gass et al. (2002) [63]^E^^,^^‡^^,^^†^	4	4	0	1.1 ± 0.3	16.7 ± 3.5	38 ± 4.6	68.7 ± 12.0	4 TETRA	*ns*	Treadmill (S-I)	moderate
Goosey-Tolfrey & Campbell (1998) [64]^‡^^,^^†^	8	7	1	2.5 ± 0.5	37.8 ± 7.9	29.9 ± 8.0	68.0 ± 11.4	7 PARA, 1 SB	*ns*	Treadmill (I)	moderate
Goosey et al. (2000) [65]^‡^^,^^†^	8	8	0	2.6 ± 0.4	43.0 ± 10.6	26 ± 8.3	61.7 ± 11.6	3 PARA, 5 SB	*ns*	WERG (S)	moderate
Goosey-Tolfrey & Tolfrey (2004) [39]^‡^^,^^†^	5	0	5	1.8 ± 0.5	33.9 ± 3.8	29 ± 7.6	52.5 ± 14.5	1 PARA, 1 AMP, 3 SB	*ns*	WERG (S)	moderate
Hooker & Wells (1992) [66]^‡^	7	6	1	2.7 ± 0.5	43.1 ± 7.4	35 ± 6.1	61.6 ± 5.7	7 PARA	specified in article	ACE (R)	moderate
Knechtle et al. (2004a) [67]^‡^^,^^†^	8	6	2	2.5 ± 0.5	41.2 ± 6.5	34.8 ± 6.3	59.6 ± 5.5	5 PARA, 2 SB, 1 PM	*ns*	Treadmill (S)	moderate
	1	1	0	1.8	32.7	51.0	56.0	1 TETRA	*ns*		
Morris (1986) [68]	1	0	1	1.4	21.1	25.0	65.5	1 PARA	80.5 km/week	ACE (R)	-
O'Connor et al. (1998) [69]^‡^^,^^†^	6	6	0	2.3 ± 0.2	36.2 ± 5.5	27.5 ± 4.9	64.1 ± 8.0	6 PARA	*ns*	WERG (S)	moderate
Perret et al. (2012) [70]^‡^	8	7	1	2.8 ± 0.7	-	32.8 ± 12.2	59.1 ± 11.0	6 PARA, 2 SB	*ns*	Treadmill (S)	moderate
Shiba et al. (2010) [71]	4	*ns*	*ns*	1.9 ± 0.4	36.9 ± 4.3	31.5 ± 9.0	52.0 ± 8.2	4 PARA	*ns*	WERG (I)	moderate
Tolfrey et al. (2001) [72]^‡^^,^^†^	16	16	0	2.4 ± 0.5	41.2 ± 9.2	28.1 ± 8.3	60.6 ± 11.0	8 PARA, 1 AMP, 6 SB, 1 PM	*ns*	WERG (S)	moderate
vd Woude et al. (2002) [26]^P^	48	3	0	0.7 ± 0.4	-	30.7 ± 8.3	61.7 ± 7.6	3 TETRA	11.3 ± 1.2 hrs/week	WERG (R)	moderate
	-	4	0	1.3 ± 0.3	-	29.5 ± 7.2	66.0 ± 9.3	4 *ns*	15.8 ± 7.2 hrs/week	WERG (R)	
	-	8	0	2.0 ± 0.3	-	31.4 ± 2.8	62.1 ± 8.8	8 *ns*	13.5 ± 3 hrs/week	WERG (R)	
	-	23	0	2.3 ± 0.4	-	27 ± 5.4	59.9 ± 11.8	23 *ns*	15.9 ± 7.3 hrs/week	WERG (R)	
	-	0	4	0.7 ± 0.2	-	29 ± 2.9	46.0 ± 4.1	4 *ns*	13.4 ± 3.3 hrs/week	WERG (R)	
	-	0	3	1.3 ± 0.1	-	26 ± 5.6	52.3 ± 10.1	3 *ns*	13.8 ± 6.8 hrs/week	WERG (R)	
	-	0	3	1.2 ± 0.2	-	23 ± 3.5	51.3 ± 8.1	3 *ns*	12.5 ± 2.5 hrs/week	WERG (R)	
Vinet et al. (1996) [57]^‡^^,^^†^	5	5	0	2.7 ± 0.6	43.6 ± 7.9	29.6 ± 4.0	63.2 ± 12.1	3 PARA, 1 SB, 1 PM	7.8 ± 3.5 hrs/week	Treadmill (S)	moderate
ARCHERY	8	7	1			-	-				
Cooper et al. (1999) [60]^‡^	4	4	0	1.9 ± 0.2	22.2 ± 2.1	44.0 ± 11.3	88.0 ± 12.9	3 PARA	*ns*	WERG (R)	moderate
	1	1	0	0.9	14.9	35	61.7	1 TETRA	*ns*		
Gass & Camp (1979) [33]	2	2	0	1.6 ± 0.3	38.3 ± 4.1	41.9 ± 2.5	-	2 PARA	11 ± 9.2 hrs/week	Treadmill (S-I)	good
Veeger et al. (1991) [50]	2	1	0	1.4	17.5	47.0	80.0	1 *ns*	*ns*	Treadmill (S-I)	moderate
	-	0	1	1.2	-	-	-	1 *ns*	*ns*	Treadmill (S-I)	
WHEELCHAIR CURLING						-	-				
Bernardi et al. (2012) [6]	10	10	0	1.8 ± 0.4	23.4 ± 7.6	42 ± 8.6	82.3 ± 29.3	1 PM, 1 LA, 8 *ns*	*ns*	ACE (R)	low
WHEELCHAIR TABLE TENNIS	8	6	2			30.1 ± 11.4	60.8 ± 8.9				
Cooper et al. (1999) [60]^P^^,^^‡^	3	1	2	1.7 ± 1.1	26.5 ± 11.5	26 ± 12.2	62.4 ± 12.0	3 PARA	*ns*	ACE (R) /WERG (R)	moderate
	1	1	0	0.96	12.2	38	78.69	1 TETRA	*ns*	WERG (R)	
Veeger et al. (1991) [50]	4	4	0	1.8 ± 0.2	30.7 ± 6.5	34 ± 11.9	60.0 ± 5.9	4 *ns*	*ns*	Treadmill (S-I)	moderate
SHOOTING	18	9	9			39.6 ± 5.5	84.8 ± 19.6				
Castle et al. (2013) [73]^E^^,^^‡^	5	3	2	1.2 ± 0.4	-	40.2 ± 1.8	69.7 ± 7.4	1 TETRA, 2 PARA, 1 SB, 1 PM	*ns*	ACE (R)	moderate
Cooper et al. (1999) [60]^P^^,^^‡^	4	2	2	1.5 ± 0.2	17.9 ± 2.7	44.8 ± 8.5	86.8 ± 20.9	4 PARA	*ns*	ACE (R) /WERG (R)	moderate
	1	0	1	0.72	7.6	52	94.7	1 TETRA	*ns*	WERG (R)	
Veeger et al. (1991) [50]	8	4	0	1.3 ± 0.3	16.3 ± 4.5	37 ± 5.1	83.0 ± 18.4	4 *ns*	*ns*	Treadmill (S-I)	moderate
	-	0	4	1.3	-	-	-	4 *ns*	*ns*	Treadmill (S-I)	
WHEELCHAIR RUGBY	114	110	4			29.9 ± 1.7	70.4 ± 3.5				
Barfield et al. (2010) [74]^†^^,^^‡^	9	9	0	1.1 ± 0.4	16.0 ± 4.7	32.7 ± 7.8	68.2 ± 15.2	9 TETRA	11.4 ± 10.4 hrs/week	ACE (R)	moderate
Domaszewska et al. (2013) [75]^‡^	14	14	0	1.3 ± 0.3	17.8 ± 5.0	34.4	72.2	14 TETRA	*ns*	ACE (R)	moderate
Goosey-Tolfrey et al. (2006) [54]^‡^^,^^†^	4	4	0	0.9 ± 0.1	12.1 ± 1.8	28.8 ± 3.2	75.0 ± 13.4	4 TETRA	*ns*	ACE (R)	moderate
Goosey-Tolfrey et al. (2014) [42]^‡^	9	9	0	1.5 ± 0.4	-	30 ± 5.0	70.6 ± 10.1	9 TETRA	13 ± 3 hrs/week	Treadmill (I)	good
Griggs et al. (2015) [43]^‡^	8	7	1	1.6 ± 0.4	-	27 ± 4.2	65.2 ± 4.4	8 TETRA	11 ± 6.4 hrs/week	Treadmill (S)	low
Leicht et al. (2012) [45]^‡^	8	8	0	1.7 ± 0.4	24.5 ± 4.9	29.2 ± 3.8	67.9 ± 6.7	8 TETRA	13.6 ± 5.6 hrs/week	Treadmill (I)	good
Morgulec-Adamowicz et al. (2011) [76]^‡^	30	7	0	1.6 ± 0.4	21.1 ± 6.3	31 ± 9.0	75.7 ± 8.2	7 TETRA	4–6 hrs/week	Treadmill (*ns*)	moderate
	-	9	0	1.8 ± 0.5	26.4 ± 6.1	31 ± 8.0	69.8 ± 12.4	9 TETRA		Treadmill (*ns*)	
	-	6	0	1.8 ± 0.5	25.6 ± 5.6	30 ± 5.0	72.5 ± 14.6	6 TETRA		Treadmill (*ns*)	
	-	8	0	2.4 ± 0.5	30.2 ± 7.2	32 ± 5.0	81.4 ± 7.8	8 TETRA		Treadmill (*ns*)	
Taylor et al. (2010) [77]	7	6	1	1.2 ± 0.5	16.9 ± 4.9	30.9 ± 5.1	70.1 ± 13.8	7 TETRA	11 ± 3 hrs/week	ACE (R)	moderate
West et al. (2013) [78]^‡^^,^^†^	7	7	0	1.3 ± 0.3	19.4 ± 3.8	31.7 ± 4.1	69.3 ± 13.5	7 TETRA	*ns*	ACE (R)	moderate
West et al. (2014a) [79]^‡^	8	7	1	1.3 ± 0.3	19 ± 2.1	29 ± 2.0	67.0 ± 15.0	8 TETRA	15 hrs/week	Treadmill (I)	moderate
West et al. (2014b) [80]^‡^	10	4	1	1.1 ± 0.2	-	27.9 ± 6.2	62.2 ± 9.2	5 TETRA	20 hrs/week	ACE (R)	moderate
	-	5	0	1.1 ± 0.2	-	30.5 ± 5.0	73.2 ± 13.3	5 TETRA		ACE (R)	

^E^ These studies are excluded in the calculation of sports disciplines means of overview Table 2, since they only provide group data which includes data of athletes with TETRA.

^P^ The data provided in these studies was only partially included of the athletes that had disabilities other than TETRA.

^‡^ Studies with data that is used in the meta-regression analyses

^†^ Studies with individual data that is used in the pooled-data multiple regression analyses

Abbreviations: *ns* not specified, *TETRA* tetraplegia, *PARA* paraplegia, *AMP* amputation, *PM* poliomyelitis, *SB* spina bifida, *LA* Les Autres, *ACE* arm crank ergometer, *WERG* wheelchair ergometer, *(S)* speed increments, *(I)* incline increments, *(S-I)* combination of speed and incline increments, *(R)* resistance increments

### 3.4 Within-sports variations

Within-sports variations in absolute and body-mass normalized VO_2peak_ values, based on CI ranges (Table 2), were relatively small in wheelchair basketball (0.4 L∙min^-1^ and 7.2 mL∙kg^-1^∙min^-1^), wheelchair racing (0.6 L∙min^-1^ and 7.4 mL∙kg^-1^∙min^-1^) and wheelchair rugby (0.4 L∙min^-1^ and 6.1 mL∙kg^-1^∙min^-1^), but above 0.6 L∙min^-1^ and 7.5 mL∙kg^-1^∙min^-1^ for the remaining sport disciplines. CI’s for absolute and body-mass normalized VO_2peak_ values could not be reported for throwing, wheelchair curling and archery, and for body-mass normalized values in Para ice hockey, as only one study with a sample size of more than two athletes was included for each of these sports disciplines.

### 3.5 Meta-regression analyses

The meta-regression analyses, based on 35 studies that provided data of 26 sub-groups and 171 individual athletes in 4 different sports disciplines (wheelchair basketball, wheelchair racing, wheelchair tennis and wheelchair rugby), resulted in the following two equations as the best predictions of absolute (1) and body-mass normalized (2) VO_2peak_ values.

$${Absolute\;VO}_{2peak}=0.93+{body\;mass}_{i}∙0.01+{\%Men}_{i}∙0.01+{\%TETRA}_{i}∙-0.01+{WERG}_{i}∙0.29+{treadmill}_{i}∙0.22$$ (1)

The factors included in Eq (1) all significantly contribute to the model (all *p* < 0.001) and explain 77% of the variance in absolute VO_2peak_. The coefficients presented in the model are unstandardized.

$${Body‑mass\;normalized\;VO}_{2peak}=40.85+{body\;mass}_{i}∙-0.12+{\%TETRA}_{i}∙-0.16+{WERG}_{i}∙4.54+{treadmill}_{i}∙4.22$$ (2)

The factors included in Eq (2) all significantly contribute to the model (all *p* < 0.001) and explain 82% of the variance in body-mass normalized VO_2peak_. The coefficients presented in the model are unstandardized

### 3.6 Pooled-data multiple regression analyses

The multiple regression analyses, based on 22 studies which provided individual data of 169 athletes in 4 different sports disciplines (wheelchair basketball, wheelchair racing, wheelchair tennis and wheelchair rugby), resulted in the following two equations as the best predictions of absolute (3) and body-mass normalized (4) VO_2peak_ values.

$${Absolute\;VO}_{2peak}=1.22+{body\;mass}_{i}∙0.02[0.25(+{female}_{i}∙-0.62[-0.25]+{TETRA}_{i}∙-1.09[-0.63]+{AMI}_{i}∙-0.29[[0.10]+{WERG}_{i}∙0.36[0.24]+{treadmill}_{i}∙0.32[0.20]$$

$$\left(F_{6,162}=52.52,p=0.00\right)$$ (3)

The factors included in Eq (3) all significantly contribute to the model (all *p* < 0.01) and explain 65% of the variance in absolute VO_2peak_. The coefficients presented in the model are unstandardized [and standardized].

$${Body‑mass\;normalizedVO}_{2peak}=49.11+{bodymass}_{i}∙-0.24[-0.26(+{female}_{i}∙-9.79[-0.24]+{TETRA}_{i}∙-16.52[-0.62]+{AMI}_{i}∙5.38[0.12]+{WERG}_{i}∙5.71[0.27]+{treadmill}_{i}∙4.54[0.18]$$

$$\left(F_{6,162}=52.50,p=0.00\right)$$ (4)

The factors included in Eq (4) all significantly contribute to the model (all *p* < 0.01) and the model explain 65% of the variance in body-mass normalized VO_2peak_. The coefficients presented in the model are unstandardized [and standardized].

## 4. Discussion

This systematic literature review aimed to (i) identify VO_2peak_ for Paralympic sitting sports, (ii) examine between-sports differences and within-sport variations in VO_2peak_ and iii) determine the influence of other factors on VO_2peak_. The main finding is that VO_2peak_ values in general reflect the sport-specific demands and the type of disability of the athletes who compete in the respective sitting sports disciplines. VO_2peak_ values range from 2.9 L∙min^-1^ and 45.6 ml∙kg^-1^∙min^-1^ in Nordic sit skiing, an endurance sport with a continuously high physical effort over sustained periods, to 1.4 L∙min^-1^ and 17.3 ml∙kg^-1^∙min^-1^ in shooting, a sport with low levels of displacement, to 1.3 L∙min^-1^ and 18.9 ml∙kg^-1^∙min^-1^ in wheelchair rugby, a sport that includes athletes with TETRA. Within-sports variation in absolute and body-mass normalized VO_2peak_ was relatively small in the sports with high sample sizes and a strong level of evidence, i.e. wheelchair basketball, wheelchair racing and wheelchair rugby, but above 0.5 L∙min^-1^ and 8 mL∙kg^-1^∙min^-1^ in all other sports. Since the VO_2peak_ values presented for each of the sports disciplines include data of athletes that differ in their sex, age, body mass, type of disability, training status and the mode they were tested in, we additionally conducted regression analyses. These analyses show that being a man, having an amputation, not being tetraplegic, testing in a wheelchair ergometer and treadmill mode, were favorable for high absolute and body-mass normalized VO_2peak_ values. Furthermore, high body mass was favourable for high absolute VO_2peak_ values and low body mass for high body-mass normalized VO_2peak_ values.

In line with our hypothesis, Nordic sit skiing, an endurance sport with continuously high physical efforts, was the Paralympic sitting sport with the highest observed absolute and body-mass normalized VO_2peak_ values. Although endurance disciplines by nature require high aerobic energy delivery, VO_2peak_ values may be particularly high in Nordic sit skiers since they compete in varying terrain, which requires both high absolute VO_2peak_ values to accompany the relatively large upper-body muscle mass required to produce sufficient power on flat terrain, as well as high body-mass normalized VO_2peak_ values to carry their body mass up inclines. The same applies to their able-bodied counterparts, standing cross-country skiers, who have shown some of the highest VO_2max_ values among Olympic athletes [9, 81, 82], although VO_2peak_ values in elite Nordic sit skiers are lower due to less active muscle mass while being tested in an upper-body mode and the adverse influence of having a disability. For example, athletes with a SCI display lower VO_2peak_ values, which is mainly related to loss of motor- and sympathetic nervous system control below the level of injury. Depending on the level and extent of injury, a SCI is associated with a range of autonomic dysregulations, which amongst other things attenuates exercise performance [83]. In fact, an inverse relationship between the level of SCI and VO_2peak_ has been found [14]. There is, however, lack of knowledge in terms of the difference in VO_2peak_ between the different disabilities represented in Paralympic sitting sports. Hutzler et al.[84] examined the aerobic power of fifty well-trained individuals with lower limb impairments including SCI, polio and amputations during arm-cranking tests in a standardized laboratory setting. It was found that individuals with high and low SCI (above and below T5, respectively) displayed lower aerobic power compared to individuals with lower limb amputations [84], which may also reflect a difference in VO_2peak_ between the SCI and other types of disability [85].

Even though not significantly different from some of the other sports disciplines, we also observed relatively high absolute VO_2peak_ values for Para ice hockey. Although this sport is characterized by short, repeated sprints requiring maximal power and speed production, aerobic capacity was shown to be highly correlated to the maintenance of sprint ability [86]. Furthermore, the high absolute VO_2peak_ values in Para ice hockey players may also be related to their large amount of upper-body muscle mass, which is required to produce power in sport-specific situations. In addition, the lack of a classification system in Para ice hockey allows athletes to perform on a high international level only if they possess good trunk control and the influence of disability is minimal, such as in athletes with a low level SCI or a lower limb amputation. Accordingly, we would have expected a low within-sports variation in the VO_2peak_ values of Para ice hockey players who are a homogenous group of Paralympic athletes with respect to gender and disabilities. However, the low number of studies and participants included in the present review in this sports discipline resulted in a limited level of evidence and wider confidence intervals for both absolute and body-mass normalized VO_2peak_ as compared to sports with a larger number of studies and participants. Therefore, the values presented might not be representative for the population of Para ice hockey players and we need to be cautious in our interpretation of VO_2peak_ values in this sport.

Wheelchair racing and handcycling are endurance sports where athletes need to sustain power over longer periods and, therefore, display relatively high body-mass normalized as well as absolute VO_2peak_ values. In fact, wheelchair racers in the present meta-analysis were found to display the second highest body-mass normalized VO_2peak_ values, which further highlights the high aerobic demands in this sports. Even though there is some variation within wheelchair racing for both absolute and body-mass normalized VO_2peak_ values, the relatively large amount of studies and participants in this sport resulted in a strong level of evidence and in narrower confidence intervals as compared to other sports disciplines with fewer studies and participants. The variance that remains can partially be explained by the classification system that allows athletes with a broad spectrum of disabilities to compete against each other in separate classes. For example, in athletes with a SCI, VO_2peak_ may be lower compared to athletes with other disabilities due to lack of sympathetic control to the paralyzed trunk and lower limbs [83] and even lower in individuals with a complete SCI above T6, who additionally lack innervation to the splanchnic area [87]. Moreover, individuals with TETRA and autonomic completeness of the injury lack sympathetic innervation to the heart and display considerably lower heart rates than athletes with other disabilities [88]. We, therefore, decided to exclude studies that included athletes with TETRA from the overview table to limit the variability in VO_2peak_. Since all these factors may influence VO_2peak_, the extent to which disability affects VO_2peak_ largely differs between Paralympic athletes, and may explain at least part of the variation in Paralympic sitting disciplines with different disability classes as compared to disciplines without. However, very small sample sizes in each of the disability classes, lack of detailed reporting on disability classes in most of the studies, and a change in the division of classes over time, prevented us from investigating the effect of disability classes on variation in VO_2peak_. Concluding from the above, differences in VO_2peak_ values between sports are fairly well reflected by the sport-specific demands and, therefore, highest in sports with continuously high physical efforts. However, they are also influenced by the heterogeneity in disabilities between athletes and the number of studies and athletes within each sports discipline, which in turn lead to differences in the magnitude of within-sport variations.

Shooting is a sport with low levels of displacement and consequently low aerobic demands. This was also reflected by the low VO_2peak_ values revealed in this sport. However, caution is warranted in the interpretation of the VO_2peak_ values in shooting despite a moderate level of evidence due to wide confidence intervals, as a result of the few studies with small sample sizes in this sports discipline. In contrast to shooting, the within-sports variation is lower and the level of evidence strong in wheelchair rugby, which increases our ability to interpret the VO_2peak_ values in this sport with more accuracy. In wheelchair rugby, a sport only eligible to athletes with impairments in both the upper and the lower limbs, the low VO_2peak_ values can be explained by the extent of the impairment. A study by West et al. [88] found that in athletes with TETRA physiological responses, including VO_2peak_, were lower as compared to other disabilities and varied based on autonomic completeness of the SCI. However, the competitiveness has increased in wheelchair rugby and athletes are training more today than previously. This is likely reflected by the increase of VO_2peak_ over time in this sport. Maybe the care (e.g. catheterization) and the access to better-adapted training facilities (e.g. endurance training equipment such as lying handbikes with supportive handles) and modalities (e.g. electrostimulation while exercising) have improved more over the last years in tetraplegic athletes than in athletes with other disabilities. Overall, VO_2peak_ values are lowest in sports with low levels of displacement or sports which include athletes with TETRA. However, the certainty in the interpretation of these values depends on the level of evidence and the within-sports variation, which are dependent on the amount of studies and sample sizes included in each sports discipline.

The VO_2peak_ values presented for each of the sports disciplines include data of athletes that differ in their sex, age, body mass, type of disability, training status and the mode they were tested in. Therefore, the effect of VO_2peak_ was considered in the meta-regression and pooled-data multiple regression analyses. These analyses indicate that being a man, having an amputation, not being tetraplegic, testing in a WERG or treadmill mode, as well as having higher or lower body mass, respectively, is favorable for high absolute and body-mass normalized VO_2peak_. The finding that being a man is beneficial for VO_2peak_ is also in line with previous studies [89, 90]. Tetraplegia may negatively influence VO_2peak_ due to a small amount of innervated muscle mass and a lack of autonomic innervation as previously discussed. In addition, that a higher body mass is beneficial for high absolute VO_2peak_ and lower body mass for high body-mass normalized VO_2peak_ was shown previously [91]. Furthermore, the finding that the WERG mode resulted in higher VO_2peak_ values compared to ACE is in line with two previous studies [92, 93], although several studies also report no differences between modes [94–97]. The reason for the clear differences between employing WERG or wheelchair treadmill testing as compared to ACE might be related to the former two modes being more sports-specific for the athletes included in the regression analyses, who all participated in wheelchair sports. Smaller coefficients for the WERG and the wheelchair treadmill mode might be expected if sitting athletes of the non-wheelchair sports are tested. The extent to which these coefficients would decrease is speculative though, as most of the latter athletes are likely using a wheelchair in at least some parts of daily life. In this context the influence of the test protocol on VO_2peak_ should also be taken into consideration. During ACE, an increased crank rate led to increases in test time and VO_2peak_ [98], whereas similar values were found in stepwise as compared to ramp type protocols [99]. Even though caution is required when drawing conclusions from the meta-regression and pooled-data multiple regression analysis, our findings provide a point of departure for understanding the influence of the above-mentioned factors on VO_2peak_ in Paralympic sitting sports athletes.

### Methodological considerations

VO_2peak_ values were provided for only 14 out of 21 Paralympic sitting sports disciplines. This is partly due to new sports disciplines being added to the Paralympic games. For example, athletes competed in para-triathlon and para-canoeing for the first time in the Paralympic games in Rio 2016, and VO_2peak_ values in these disciplines are hence missing. So far, the only sports where a considerable number of VO_2peak_ values was provided with a strong level of evidence and we can hence conclude with more certainty are wheelchair basketball, wheelchair racing and wheelchair rugby with 234, 205 and 114 included athletes, respectively. Thus, more studies and bigger data pools established through international collaborations are required. Alternatively, systematically combining the results of multiple studies in a literature review and meta-analysis can compensate for the small sample sizes in original studies in the Paralympic field. However, to allow for more valid analyses, future studies are encouraged to provide sufficient detail on outcome measures in their abstracts and to provide individual, more detailed anthropometric and training data of their athletes. Furthermore, possible changes in the demands of the sports and improvements in performance and physiological capacity over the years should further be elucidated in future studies.

The studies included in the current review vary widely in terms of test equipment, such as ergospirometers and weighing scales, as well as the test mode, warm-up procedure and test protocol (stepwise increments in resistance, speed, incline or a combination of speed and incline). The effect of variations in these factors on upper-body VO_2peak_ in athletes with a disability remains unclear. To enable a more valid comparison of findings between studies, future studies should aim at providing enough details on the above mentioned factors and on finding standardized criteria for determination of VO_2peak_ in upper-body exercise modes.

In case of the present literature review, the consequences of publication bias are not only related to being able to publish data with significant findings and/or positive findings. It may also be related to the nature of elite sports where many countries test their best athletes without publishing this information. The reason may be two-fold, since giving away interesting information may help competitors and/or simply because publishing would be too resource demanding. Furthermore, data of Paralympic athletes might not be published because of a too low number of participants included to run statistical analyses on the data or due to the tested athletes not being considered elite, which was especially the case a few decades ago. Therefore, the average VO_2peak_ values presented here might not fully reflect the VO_2peak_ of medal-winning elite athletes in many of the sports. However, we are confident that in the sports with a strong level of evidence (wheelchair basketball, wheelchair racing and wheelchair rugby), the ranges provided for the VO_2peak_ values reflect the aerobic capacity of athletes in the respective sports. Although we limited the effect of publication bias by excluding articles with a complete overlap of data, we cannot exclude that duplicate data of individual athletes is included in this review. The likelihood of publication bias is illustrated by the fact that 15 of the articles included are from the research-group of Goosey-Tolfrey et al.[36, 39–43, 45, 46, 53–55, 64, 65, 72, 79]. Additionally, VO_2peak_ was a secondary measure in many of the reviewed studies. We, therefore, screened a large amount of abstracts of studies that did not directly mention VO_2peak_ in their title in order to reduce the possibility to miss articles that could have fit our inclusion criteria.

A limitation in the present review is that information on training status, which is known to influence VO_2peak_, was missing in a considerable amount of studies.

In the absence of a valid allometric scaling method for athletes with different disabilities and across various sports, we provided only absolute and body-mass normalized values in this review. However, we refer to the studies of Batterham et al. [100] for the challenges surrounding the units in which VO_2peak_ is presented and to Goosey-Tolfrey et al. [17] for the only study on scaling of VO_2peak_ values in athletes with a disability.

## 5. Conclusion

In the current review, VO_2peak_ values for Paralympic sitting sports were systematically reported in 14 out of 21 possible sitting sports disciplines. Of these, VO_2peak_ was highest in the typical endurance sports and lowest in sports with low levels of displacement and in those including athletes with TETRA. However, the only sports where a sufficient number of VO_2peak_ values are combined with a strong level of evidence, thereby allowing us to conclude with more certainty, are wheelchair basketball, wheelchair racing and wheelchair rugby. In contrast, VO_2peak_ values should be interpreted carefully in disciplines with limited level of evidence or with only one study mean and in disciplines with large within-sports variations. Large within-sports variation was found in sports with few included studies and corresponding low sample sizes. The VO_2peak_ values presented for each of the sports disciplines include data of athletes that differ in their sex, age, body mass, type of disability, training status and the mode they were tested in. The influence of these factors on VO_2peak_ was investigated in regression analyses, which indicated that–in wheelchair basketball, wheelchair racing, wheelchair tennis and wheelchair rugby athletes–being a man, having an amputation, not being tetraplegic, testing in a wheelchair ergometry or treadmill mode, was beneficial for attaining high absolute or body-mass normalized VO_2peak_ values. Furthermore, high body mass was favourable for high absolute VO_2peak_ values and low body mass for high body-mass normalized VO_2peak_ values. In general, the practical applications of this review are limited due to most sports disciplines having large within-sports variations in VO_2peak_, a limited level of evidence or including only one study mean. Based on the findings of this study and as a take-home message for future studies, we encourage the use of standardized determination criteria for reaching VO_2peak_, and the inclusion of more detailed information on training status, sex, age, body mass, type of disability and testing mode, as well as larger study samples from international collaborations.

## Supporting information

S1 Fig Boolean search string. Boolean search string with combined synonyms and MeSH terms (the latter only for the search in PubMed), which was entered into the data bases.
(PDF)

S1 Table Adjusted black and downs checklist. (PDF)

S2 Table Quality scores of the 57 included studies. Scoring of quality items for each of the studies included in this systematic literature review on peak oxygen uptake in Paralympic sitting sports.
(PDF)

S3 Table PRISMA 2009 checklist. (PDF)

S1 Excel file Extracted and analyzed data of this systematic literature review on peak oxygen uptake in Paralympic sitting sports. (XLSX)
