# Correction: Peak oxygen uptake in Paralympic sitting sports: A systematic literature review, meta- and pooled-data analysis

**DOI:** 10.1371/journal.pone.0200326

**Published:** 2018-07-03

**Authors:** The PLOS ONE Staff

There are errors in Eqs 3 and 4. Please see the correct equations below. The publisher apologizes for the errors.

$$\begin{array}{l} {\mathrm{Absolute}\;\mathrm{VO}}_{2\mathrm{peak}}=1.22+{\mathrm{body}\;\mathrm{mass}}_{i}∙0.02\;[0.25]+\;{\mathrm{female}}_{i}∙-0.62\;[-0.25]+{\mathrm{TETRA}}_{i\;}∙\;-1.09\;[-0.63]+\;{\mathrm{AMP}}_{i}\;∙0.29\;[0.10]+{\mathrm{WERG}}_{i}\;∙0.36\;[0.24]+{\mathrm{treadmill}}_{i}∙0.32\;[0.20]\\ \;\left(F_{6,162}=52.52,p=0.00\right) \end{array}$$ (3)

$$\begin{array}{l} {\mathrm{Body-mass}\;\mathrm{normalized}\;\mathrm{VO}}_{2\mathrm{peak}}=49.11+{\mathrm{body}\;\mathrm{mass}}_{i}∙-0.24\;[-0.26]+\;{\mathrm{female}}_{i}∙-9.79\;[-0.24]+{\mathrm{TETRA}}_{i\;}∙\;-16.52\;[-0.62]+\;{\mathrm{AMP}}_{i}\;∙5.38\;[0.12]+{\mathrm{WERG}}_{i}\;∙5.71\;[0.27]+{\mathrm{treadmill}}_{i}∙4.54\;[0.18]\\ \;\left(F_{6,162}=52.50,p=0.00\right) \end{array}$$ (4)
