# Supplementary material for: Peak oxygen uptake in Paralympic sitting sports: A systematic literature review, meta- and pooled-data analysis
Source: PLoS One. 2018 Feb 23;13(2):e0192903. doi: 10.1371/journal.pone.0192903 (PMC5825058; doi:10.1371/journal.pone.0192903)
Supplement: S1 Fig — Boolean search string with combined synonyms and MeSH terms (the latter only for the search in PubMed), which was entered into the data bases. (PDF) [file pone.0192903.s001.pdf]

S1 Fig. Boolean search string

Boolean search string with combined synonyms and MeSH terms (the latter only for the search in PubMed), which was entered into the data bases.

(“physical endurance” [MeSH] OR “oxygen uptake” OR power OR “upper body” OR “upper limb” OR “upper extremity” [MeSH] OR oxygen OR “aerobic endurance” OR “aerobic capacity” OR “aerobic power” OR “aerobic performance” OR “exercise capacity” OR “exercise physiology” OR “physical performance” OR (cardiorespiratory AND (response OR responses)) OR “arm crank” OR “arm cranking” OR ergometer OR ergometry OR “poling ergometer” OR VO2max OR VO2peak) AND ((para OR Paralympic* OR wheelchair*) OR ((wheelchair* OR sitting) AND (athlete OR athletes)) OR (sitting AND (sport OR sports)) OR (wheelchair* AND (sport OR sports)) OR (wheelchair* AND (athlete OR athletes)) OR ((archery OR archer OR archers) AND (para OR Paralympic* OR wheelchair*)) OR (boccia AND (para OR Paralympic* OR wheelchair*)) OR (basketball AND (para OR Paralympic* OR wheelchair*)) OR (tennis AND (para OR Paralympic* OR wheelchair*)) OR (“table tennis” AND (para OR Paralympic* OR wheelchair*)) OR (badminton AND (para OR Paralympic* OR wheelchair*)) OR (rugby AND (para OR Paralympic* OR wheelchair*)) OR ((fencer* OR fencing) AND (para OR Paralympic* OR wheelchair*)) OR (wheelchair AND (racer* OR racing)) OR (track AND wheelchair*) OR “wheelchair marathon” OR (hand AND (cycling OR cyclist*)) OR (hand AND (biking OR biker*)) OR handbik* OR handcycl* OR (volleyball AND (para OR Paralympic* OR sitting)) OR ((triathlon OR triathlete*) AND (para OR Paralympic*)) OR ((rower* OR rowing) AND (para OR Paralympic*)) OR ((sit OR sitting) AND (ski OR skier*)) OR (((alpine AND ski) OR (alpine AND skiing) OR (alpine AND skier) OR (alpine AND skiers)) AND (para OR Paralympic*)) OR (((Nordic AND ski) OR (Nordic AND skiing) OR (Nordic AND skier) OR (Nordic AND skiers)) AND (para OR Paralympic*)) OR ((biathlon OR biathlete*) AND (para OR Paralympic* OR sitting)) OR “sledge hockey” OR (wheelchair* AND (curling OR curler*)) OR (“shot put” AND sitting) OR (javelin AND (para OR Paralympic* OR sitting)) OR (shoot* AND (para OR Paralympic* OR sitting)) OR ((canoeing OR canoeist*) AND (para OR Paralympic*)) OR ((sailing OR sailor*) AND (para OR Paralympic*)) OR (equestrian* AND (para OR Paralympic*)))
