# Supplementary material for: Peak oxygen uptake in Paralympic sitting sports: A systematic literature review, meta- and pooled-data analysis
Source: PLoS One. 2018 Feb 23;13(2):e0192903. doi: 10.1371/journal.pone.0192903 (PMC5825058; doi:10.1371/journal.pone.0192903)
Supplement: S1 Table — (PDF) [file pone.0192903.s002.pdf]

S1 Table. Adjusted Downs and Black Quality Assessment Checklist [1]

**Reporting**					
1. *Is the hypothesis/aim/objective of the study clearly described?* This item is only rated as a Yes, if both aim/purpose and hypothesis are described. In case the study design does not allow any hypothesis or the direction of the study is so novel that no prior hypothesis can be formed, and this is made clear in the introduction, the item should be rated as a Yes.	**Yes**		**No**	**U**	
2. *Are the main outcomes to be measured clearly described in the Introduction or Methods section?* If the main outcomes are first mentioned in the Results section, the question should be answered No.	**Yes**		**No**	**U**	
3. *Are the characteristics of the participants included in the study clearly described?* In cohort studies and trials, inclusion and/or exclusion criteria should be given. In case-control studies, a case-definition and the source for controls should be given. The type of disability should be clearly described. For athletes with a spinal cord injury it has to be indicated (at least) if they are tetraplegic or paraplegic for this item to be rated with a Yes. If it is clear from the inclusion criteria which participants are excluded, rate this item as a Yes. If this is not clear and the study does not mention any exclusion criteria, rate this items as a No.	**Yes**		**No**	**U**	
5. *Are the distributions of principal confounders in each group of subjects to be compared clearly described?* The main confounders of our study are: sex, age, disability, training status, body-mass. Secondary confounders are: testing moment during the season and test mode. For scoring 2 points all five main confounders need to be described. The criteria for describing the disability in enough details are the same as for item 3. For scoring 1 point four of the five main confounders plus one secondary confounder need to be mentioned.	**Yes (2)**	**Yes (1)**	**No**	**U**	
6. *Are the main findings of the study clearly described?* Simple outcome data (including denominators and numerators) should be reported for all major findings so that the reader can check the major analyses and conclusions. In the case of this review, the number of participants has to be given, as well the VO_{2peak} values. (This question does not cover statistical tests which are considered below).	**Yes**		**No**	**U**	
7. *Does the study provide estimates of the random variability in the data for the main outcomes?* In non-normally distributed data the inter-quartile range of results should be reported. In normally distributed data the standard error, standard deviation or confidence intervals should be reported. If the distribution of the data is not described, it must be assumed that the estimates used were appropriate and the question should be answered Yes. If no mean and SD are calculated and only individual items are provided, rate this item as a No.	**Yes**		**No**	**U**	
**External Validity**<br>All the following criteria attempt to address the representativeness of the findings of the study and whether they may be generalized to the population from which the study subjects were derived.					
11. *Were the subjects asked to participate in the study representative of the entire population from which they were recruited?* The study must identify the source population for participants and describe how the participants were selected. Participants would be representative if they comprised the entire source population, an unselected sample of consecutive participants, or a random sample. Random sampling is only feasible where a list of all members of the relevant population exists. Where a study does not report the proportion of the source population from which the patients are derived, the question should be answered as unable to determine. The source population in our review is defined as athletes with a disability in the respective sports.	**Yes**		**No**	**U**	**N/A**
12. *Were those subjects who were prepared to participate representative of the entire population from which they were recruited?* The proportion of those asked who agreed should be stated. Validation that the sample was representative would include demonstrating that the distribution of the main confounding factors was the same in the study sample and the source population.	**Yes**		**No**	**U**	**N/A**
**Internal validity – bias**					
20. *Were the VO_{2peak} measure used accurate (valid and reliable)?* Studies have to report on the reliability and validity of the test equipment, test mode and if people with a disability (note: here we do not refer to athletes) were tested. For studies which refer to other work that demonstrates the outcome measures are accurate, the question should be answered as Yes. Both, pilot testing or referring to the work of others are sufficient to rate this item as a Yes. Referring to reliability and variability estimates performed on able-bodied participants is considered sufficient and the item will be rate as a Yes.	**Yes**		**No**	**U**	**N/A**
**Internal validity - confounding (selection bias)**					
21. *Were the participants in different intervention groups (trials and cohort studies) or were the cases and controls (case-control studies) recruited from the same population?* The question should be answered unable to determine for cohort and case control studies where there is no information concerning the source of patients included in the study. The source population in this review is defined as athletes with a disability in the respective sports.	**Yes**		**No**	**U**	**N/A**

22. *Were study participants in different intervention groups (trials and cohort studies) or were the cases and controls (case-control studies) recruited over the same period of time?* For a study which does not specify the time period over which patients were recruited, the question should be answered as unable to determine.	**Yes**	**No**	**U**	**N/A**
25. *Was there adequate adjustment for confounding in the analyses from which the main findings were drawn?* This item should be rated as a Yes, if the criteria for verification of maximal effort are explicitly stated. Verification of maximal effort should at least contain two of the following five minimum criteria: 1) respiratory exchange ratio (RER) of 1.05 or higher, 2) a concentration of lactate in blood ($[La^-]_b$) of 7 mmol/liter or greater, and 3) a subjective rating of perceived exertion (RPE) with a BORG scale score of 15 or higher, 4) no increase in VO_2 despite further increases in intensity or 5) reaching a maximal heart rate within 10 beats/min of an individual's age-predicted maximum (calculated as 220 – age). Alternatively, the verification of maximal effort is also considered to be achieved in case a verification test was performed. Any studies which did not report on the verification of maximal effort or which include criteria below the above described, should be rated with a No. Deviating criteria should be specifically noted in the comments box provided to the right.	**Yes**	**No**	**U**	**N/A**

Yes (1 point), No (0 points), U = unable to determine (0 points), N/A = not applicable (question excluded from quality analysis)

References

1. Downs SH, Black N. The feasibility of creating a checklist for the assessment of the methodological quality both of randomised and non-randomised studies of health care interventions. J Epidemiol Community Health. 1998;52(6):377-84.
