# Supplementary material for: Peak oxygen uptake in Paralympic sitting sports: A systematic literature review, meta- and pooled-data analysis
Source: PLoS One. 2018 Feb 23;13(2):e0192903. doi: 10.1371/journal.pone.0192903 (PMC5825058; doi:10.1371/journal.pone.0192903)
Supplement: S2 Table — Scoring of quality items for each of the studies included in this systematic literature review on peak oxygen uptake in Paralympic sitting sports. (PDF) [file pone.0192903.s003.pdf]

S2 Table. Quality scores of the 57 included studies

Scoring of quality items for each of the studies included in this systematic literature review on peak aerobic capacity between and within Paralympic sitting sports

	1	2	3	5	6	7	11	12	20	21	22	25	Points scored	Number of applicable items	Percentage of points scored	Methodological quality
Barfield et al. (2010) [74]	no	yes	yes	yes (2)	yes	yes	u	no	u	yes	u	no	7	13	54	moderate
Bernardi et al. (2010) [2]	yes	yes	yes	yes (1)	yes	yes	u	no	u	yes	yes	yes	9	13	69	good
Bernardi et al. (2012) [6]	yes	yes	no	no	yes	yes	u	no	u	yes	u	no	5	13	38	low
Bhambhani et al. (1995) [58]	yes	yes	yes	yes (1)	yes	yes	u	no	u	yes	u	no	7	13	54	moderate
Bhambhani et al. (2012) [28]	no	yes	yes	yes (1)	yes	yes	u	no	yes	yes	u	no	7	13	54	moderate
Bloxham et al. (2001) [34]	yes	yes	no	no	yes	no	u	no	u	yes	u	yes	5	13	38	low
Campbell et al. (2004) [25]	no	yes	yes	yes (1)	yes	yes	u	no	yes	yes	u	no	7	13	54	moderate
Castle et al. (2013) [73]	yes	yes	yes	yes (1)	yes	yes	u	no	u	yes	u	no	7	13	54	moderate
Cooper et al. (1992) [59]	no	yes	yes	yes (2)	yes	yes	u	no	u	yes	yes	no	8	13	62	moderate
Cooper et al. (1999) [60]	yes	yes	yes	no	yes	yes	u	no	u	yes	u	yes	7	13	54	moderate
Coutts & Stogryn (1987) [61]	no	yes	yes	yes (1)	yes	no	u	no	u	yes	yes	no	6	13	46	moderate
Coutts et al. (1990) [35]	no	yes	yes	no	yes	yes	u	no	u	yes	yes	no	6	13	46	moderate
Crews et al. (1982) [62]	no	yes	yes	yes (2)	yes	no	u	no	u	yes	u	no	6	13	46	moderate
Croft et al. (2010) [36]	no	yes	yes	yes (2)	yes	yes	u	yes	u	yes	u	yes	9	13	69	good
de Lira et al. (2010) [37]	yes	yes	yes	yes (1)	yes	yes	u	no	u	yes	u	no	7	13	54	moderate
Diaper & Goosey-Tolfrey (2009) [53]	no	yes	yes	yes (1)	yes	N/A	N/A	N/A	u	N/A	N/A	yes	5	8	63	-
Domaszewska et al. (2013) [75]	no	yes	yes	yes (1)	yes	yes	u	no	u	yes	u	no	6	13	46	moderate
Dwyer & Davis (1998) [38]	no	yes	no	no	yes	yes	u	no	u	yes	u	no	4	13	31	low
Fischer et al. (2014) [30]	yes	yes	yes	yes (2)	yes	yes	u	no	u	yes	u	no	8	13	62	moderate
Gass & Camp (1979) [33]	yes	yes	yes	yes (2)	yes	yes	u	no	u	yes	yes	no	9	13	69	good
Gass et al. (2002) [63]	yes	yes	yes	yes (1)	yes	yes	u	no	u	yes	u	no	7	13	54	moderate
Goll et al. (2015) [52]	no	yes	no	no	yes	yes	u	no	u	yes	u	yes	5	13	38	low
Goosey & Campbell (1998) [64]	no	yes	yes	yes (1)	yes	yes	u	no	yes	yes	u	no	7	13	54	moderate
Goosey et al. (2000) [65]	yes	yes	yes	yes (1)	yes	yes	u	no	u	yes	u	no	7	13	54	moderate
Goosey-Tolfrey & Tolfrey (2004) [39]	no	yes	yes	yes (1)	yes	yes	u	no	u	yes	u	yes	7	13	54	moderate

Goosey-Tolfrey et al. (2005) [40]	no	yes	yes	yes (2)	yes	yes	u	no	u	yes	yes	yes	9	13	69	good
Goosey-Tolfrey et al. (2006) [54]	no	yes	yes	yes (1)	yes	yes	u	no	u	yes	u	no	6	13	46	moderate
Goosey-Tolfrey et al. (2008) [55]	yes	yes	yes	yes (1)	yes	yes	u	no	u	yes	u	no	7	13	54	moderate
Goosey-Tolfrey & Tolfrey (2008) [41]	no	yes	yes	yes (1)	yes	yes	u	no	u	yes	u	yes	7	13	54	moderate
Goosey-Tolfrey et al. (2014) [42]	no	yes	yes	yes (2)	yes	yes	u	no	yes	yes	u	yes	9	13	69	good
Griggs et al. (2015) [43]	no	yes	yes	yes (2)	yes	yes	u	no	u	yes	u	no	7	13	54	moderate
Hooker & Wells (1992) [66]	no	yes	yes	yes (2)	yes	yes	u	no	u	yes	u	no	7	13	54	moderate
Knechtle & Kopfli (2001) [44]	no	yes	yes	yes (1)	yes	yes	u	no	u	yes	u	no	6	13	46	moderate
Knechtle et al. (2004b) [31]	no	yes	yes	yes (1)	yes	yes	u	no	u	yes	u	no	6	13	46	moderate
Knechtle et al. (2004a) [67]	no	yes	yes	yes (1)	yes	yes	u	no	u	yes	u	no	6	13	46	moderate
Leicht et al. (2012) [45]	yes	yes	yes	yes (2)	yes	yes	u	no	u	yes	u	yes	9	13	69	good
Leicht et al. (2014) [46]	yes	yes	yes	yes (2)	yes	yes	u	no	u	yes	u	no	8	13	62	moderate
Lovell et al. (2012) [32]	no	yes	yes	yes (2)	yes	yes	u	no	u	yes	u	no	7	13	54	moderate
Morgulec-Adamowicz et al. (2011) [76]	yes	yes	yes	yes (2)	yes	yes	u	no	u	yes	u	no	8	13	62	moderate
Morris (1986) [68]	no	yes	yes	yes (2)	yes	N/A	N/A	N/A	u	N/A	N/A	no	5	8	63	-
O'Connor et al. (1998) [69]	yes	yes	yes	yes (1)	yes	yes	u	no	u	yes	u	no	7	13	54	moderate
Perret et al. (2012) [70]	yes	yes	yes	yes (1)	yes	yes	u	no	u	yes	u	no	7	13	54	moderate
Rotstein et al. (1994) [47]	no	yes	yes	yes (1)	yes	yes	u	no	u	yes	u	no	6	13	46	moderate
Roy et al. (2006) [56]	no	yes	yes	yes (2)	yes	yes	u	no	u	yes	u	yes	8	13	62	moderate
Sandbakk et al. (2014) [81]	yes	yes	no	yes (1)	yes	yes	u	no	u	yes	u	yes	7	13	54	moderate
Schmid et al. (1998) [48]	no	yes	yes	yes (2)	yes	yes	u	no	u	yes	u	no	7	13	54	moderate
Shiba et al. (2010) [71]	no	yes	yes	yes (1)	yes	yes	u	no	u	yes	u	yes	7	13	54	moderate
Taylor et al. (2010) [77]	no	yes	yes	yes (2)	yes	yes	u	no	u	yes	u	no	7	13	54	moderate
Tolfrey et al. (2001)[72]	no	yes	yes	yes (1)	yes	yes	u	no	u	yes	u	yes	7	13	54	moderate
van der Woude et al. (2002) [26]	no	yes	yes	yes (2)	yes	yes	u	no	u	yes	u	no	7	13	54	moderate
Vanlandewijck et al. (1994) [49]	no	yes	yes	yes (2)	yes	yes	u	no	u	yes	u	no	7	13	54	moderate
Veeger et al. (1991) [50]	no	yes	yes	yes (1)	yes	yes	u	no	u	yes	yes	no	7	13	54	moderate
Vinet et al. (1996) [57]	no	yes	yes	yes (2)	yes	yes	u	no	u	yes	u	no	7	13	54	moderate
West et al. (2013) [78]	yes	yes	yes	yes (1)	yes	yes	u	no	u	yes	u	no	7	13	54	moderate
West et al. (2014) [80]	yes	yes	yes	yes (2)	yes	yes	u	no	u	yes	u	no	8	13	62	moderate
West et al. (2014) [79]	yes	yes	yes	yes (2)	yes	yes	u	no	u	yes	u	no	8	13	62	moderate
Zacharakis et al. (2012) [51]	yes	yes	yes	yes (1)	yes	yes	u	no	u	yes	u	no	7	13	54	moderate
